# Prevalence and risk factors of depressive symptoms among 140,259 college students during the COVID-19 epidemic normalization in China: A cross-sectional survey

**DOI:** 10.3389/fpubh.2022.936700

**Published:** 2022-08-24

**Authors:** Xiaoyu Luo, Qingqing Xu, Keliang Fan, Juan Wang, Dandan Wei, Xian Wang, Xiaomin Lou, Hualiang Lin, Chongjian Wang, Cuiping Wu, Zhenxing Mao

**Affiliations:** ^1^Department of Epidemiology and Biostatistics, College of Public Health, Zhengzhou University, Zhengzhou, China; ^2^Department of Epidemiology, School of Public Health, Sun Yat-sen University, Guangzhou, China

**Keywords:** depressive symptoms, COVID-19, college students, factors, China

## Abstract

**Objective:**

College students are one of the most vulnerable populations to the COVID-19 pandemic's mental health effects. During the coronavirus disease 2019 (COVID-19) outbreak, we wanted to see how common depressive symptoms were among college students and what factors contributed to that.

**Methods:**

Between 21 and 27 May 2021, 140,259 college students from three cities in Henan Province, China, were involved. The Patient Health Questionnaire-9 was used to determine depressive symptoms (PHQ-9). Multiple logistic regression analysis was used to calculate odds ratios (ORs) and 95% CIs for potential depressive symptom factors.

**Results:**

Mild depressive symptoms and above are present in 21.12% of college students. Women had a higher prevalence of mild depressive symptoms than men (61.38 vs. 59.75%), and depressive symptoms were most prevalent among rural students and least prevalent among city students (21.44 vs. 20.29%). Participants with depressive symptoms are also more likely to have a poor-behavioral status. From none-to-severe depressive symptoms, 78.88, 15.78, 2.80, 1.67, and 0.88% had them. Gender, residential location, and behavioral status were found to be associated with depressive symptoms after adjusting for potential confounders.

**Conclusion:**

This cross-sectional study identified the factors that influence the prevalence of depression in college students. It found that the government should pay more attention to mental health issues affecting college students in combating the COVID-19 epidemic normalization.

## Introduction

A new-type coronavirus, which has been identified in December 2019 (1), has caused global health concerns due to its devastating impact (2, 3). The imported cases from abroad have been controlled in China at this stage. The epidemic situation in China is sporadic on the whole, and there are occasional small-scale epidemic situations in some parts. In response to the outbreak of the virus, the Chinese government has implemented various measures to prevent the spread of the disease. These include the suspension of public transportation and the closure of non-essential leisure and entertainment establishments (4). A longitudinal population-based study shows that symptoms of anxiety and depression are significant during COVID-19 and increase during lockdown (5, 6). At the same time, some studies have shown that being isolated from others can lead to depressive symptoms. The link between isolation and anxiety has been linked to a variety of mental health issues (7, 8). It has been known that students who are forced to stay at home and are socially isolated are more prone to experiencing higher levels of depression (9, 10).

The depressive symptom is a common mental health disorder that affects the mental health of the general population. It has a worldwide prevalence of 17.3% when using self-reporting instruments to assess depression and is considered to be a mental health disorder (11). In comparison to their counterparts throughout the world, college students have greater rates of mental disorders such as depressive symptoms (12), which can cause horrible feelings such as fear, inadequacy, and wrath, and also psychological and physical morbidities (13–15). Loneliness and illness management measures increase the risk of depression in previously healthy children and adolescents, according to a comprehensive systematic evaluation of data from over 50,000 children and adolescents in 63 research (16, 17). It is clear that the COVID-19 pandemic's direct and indirect psychological and social consequences are pervasive (18–21).

Most of the current literature on the psychological impact of COVID-19 has focused on health workers, patients, and children the general population (22–26). Studies have shown that COVID-19 exposure can lead to high levels of anxiety and depressive symptoms among healthcare workers (27), which raises widespread concern. Of note, college students are at the age of high risk for the onset of mental illness (28, 29). Despite growing evidence of mental health complications from COVID-19 among middle- and high-school students in China (30), there is sparse evidence of psychological or mental health effects of the COVID-19 pandemic on college students, and large sample survey evidence for this group of college students is still very limited. In the face of COVID-19 outbreak, expansion in China and other parts of the world, this study assessed the prevalence of depressive symptoms among college students and identified potential risk and protective factors contributing to depressive symptoms to assist government agencies and healthcare professionals in safeguarding the school's psychological wellbeing.

## Methods

### Study participants

The cross-sectional study is to investigate the impact of the COVID-19 pandemic on the college student depressive symptoms by using an online questionnaire through an online survey platform (“SurveyStar,” Changsha Ranxing Science and Technology, Shanghai, China) during 21–27 May 2021. In the three cities in Henan Province, China, college students were selected and invited to participate in the survey using a cluster sampling method. In total, 148,999 participants were recruited. For quality control, we excluded the data of participants aged <18 years or aged >25 years or those who took ≤ 100 s to fully respond to the questions (*n* = 8,740). After this exclusions process, a total of 140,259 participants, aged 18–25 years, were included in this analysis.

The study protocol was approved by the Ethics Committee of Zhengzhou University (ZZUIRB2021-118).

### Data collection

To collect sociodemographic data, a standard questionnaire was devised (age, gender, grade, and residential location). The behavioral status about COVID-19 includes “Has the number of handwashing increased significantly after the outbreak,” “The situation of wearing masks when going out after the outbreak,” as well as mental state (worry and fear levels) and depressive symptoms. City, rural, and county-level cities were used to classify residential locations. The worry and fear levels were divided into five levels (extremely, very, somewhat, not so, and not at all) based on 5-point Likert scale (31). To examine depressive symptoms, the Patient Health Questionnaire-9 (PHQ-9) was employed (32). The PHQ-9 is based on the Diagnostic and Statistical Manual of Mental Disorders, 4th Edition's diagnostic criteria for depressed symptoms (DSM-IV). The PHQ-9's psychometric qualities have already been established in the Chinese populations and developing-country medical settings (32–34). On a 27-point scale, participants were asked how often nine symptoms had emerged in their lives in the previous two weeks: 0 = “not at all,” 1 = “several days,” 2 = “more than half the days,” 3 = “nearly every day.” The severity of depressive symptoms was scored as follows: 0–4 for no depression symptoms, 5–9 for mild, 10–14 for moderate, 15–19 for moderately severe, and 20–27 for severe depressed symptoms (35). A cutoff of 10 or more is reported as diagnostic (35).

### Statistical analysis

Continuous data were presented as mean ± standard deviation (SD) and compared using the *t*-test, while categorical variables were presented as frequencies (%) and the significance of categorical variable differences was determined using the chi-squared test. The odds ratios (ORs) and 95% CIs of depressed symptoms were calculated using a logistic regression model. Multivariable adjustment modeling was performed: Model 1 was the crude model. Model 2 was adjusted for gender, residential location, worried level, and fear level. Statistical Package for the Social Sciences (SPSS) (version 26) was used for all statistical analyses, with *P* values of <0.05 indicating statistical significance. Imputation or other substitution procedures were not employed, and only respondents who provided complete data were included in the analysis.

## Result

### Basic characteristics of participants

In total, 140,259 participants (aged 18–25 years), which include 70,123 men and 70,136 women, were invited in to participate in the survey from 27 May to 27 May. The characteristics of the participants were listed in Table 1 along with their relationships with depressed symptom status. The study population contained 6,384 cases of depressive symptoms (4.54%). Participants with depressive symptoms had different proportions of age, gender, residence location, and behavioral status than those without depressive symptoms (all *P* values of <0.05).

**Table 1:** Characteristics of the study participants by depression status.

**Characteristics**	**All participants**	**No-depression**	**Depression**	***P* value**
	**n = 140,259**	**n = 133,885**	**n = 6,374**	
**Age (years)**	20.43 ± 4.57	20.43 ± 4.57	20.47 ± 4.53	<0.001
**Gender (%)**				<0.001
Male	70,123 (50.00)	66,717 (49.83)	3,406 (53.44)	
Female	70,136 (50.00)	67,168 (50.17)	2,968 (46.56)	
**Residential location (%)**				<0.001
City	29,962 (21.36)	28,581 (21.35)	1,381 (21.67)	
Rural	78,604 (56.04)	75,078 (56.08)	3,526 (55.32)	
Country-level city	31,693 (22.60)	30,226 (22.58)	1,467 (23.02)	
**Worried level (%)**				<0.001
High	69,154 (49.31)	65,356 (48.82)	3,798 (59.58)	
Moderate	43,235 (30.83)	41,701 (31.15)	1,534 (24.07)	
Low/none	27,870 (19.87)	26,828 (20.04)	1,042 (16.35)	
**Fear level (%)**				<0.001
High	38,599 (27.52)	35,862 (26.78)	2,737 (42.94)	
Moderate	62,507 (44.57)	60,229 (44.99)	2,278 (35.74)	
Low/none	39,153 (27.92)	37,794 (28.23)	1,359 (21.32)	
**Has the number of handwashing increased significantly after the outbreak (%)**				<0.001
Yes	133,858 (95.44)	12,8197 (95.75)	5,668 (88.78)	
No	6,401 (4.56)	5,688 (4.25)	716 (11.22)	
**The situation of wearing masks when going out after the outbreak (%)**				<0.001
Always wear a mask when you go out	100,010 (71.30)	96,961 (71.67)	4,061 (63.61)	
Wear masks when going to crowded places or taking public transport	38,537 (27.48)	36,512 (27.27)	2,029 (31.78)	
Wear masks occasionally	1,469 (1.05)	1,250 (0.93)	223 (3.49)	
Do not wear masks	243 (0.17)	172 (0.13)	71 (1.11)	

*Data were presented as mean (SD) normal distribution continuous variables and numbers (percentages) for categorical variables; P values were calculated using student's t-test and chi-square. Compared with no-depression, P <0.05*.

### Prevalence of depressive symptoms

**Figure 1:**
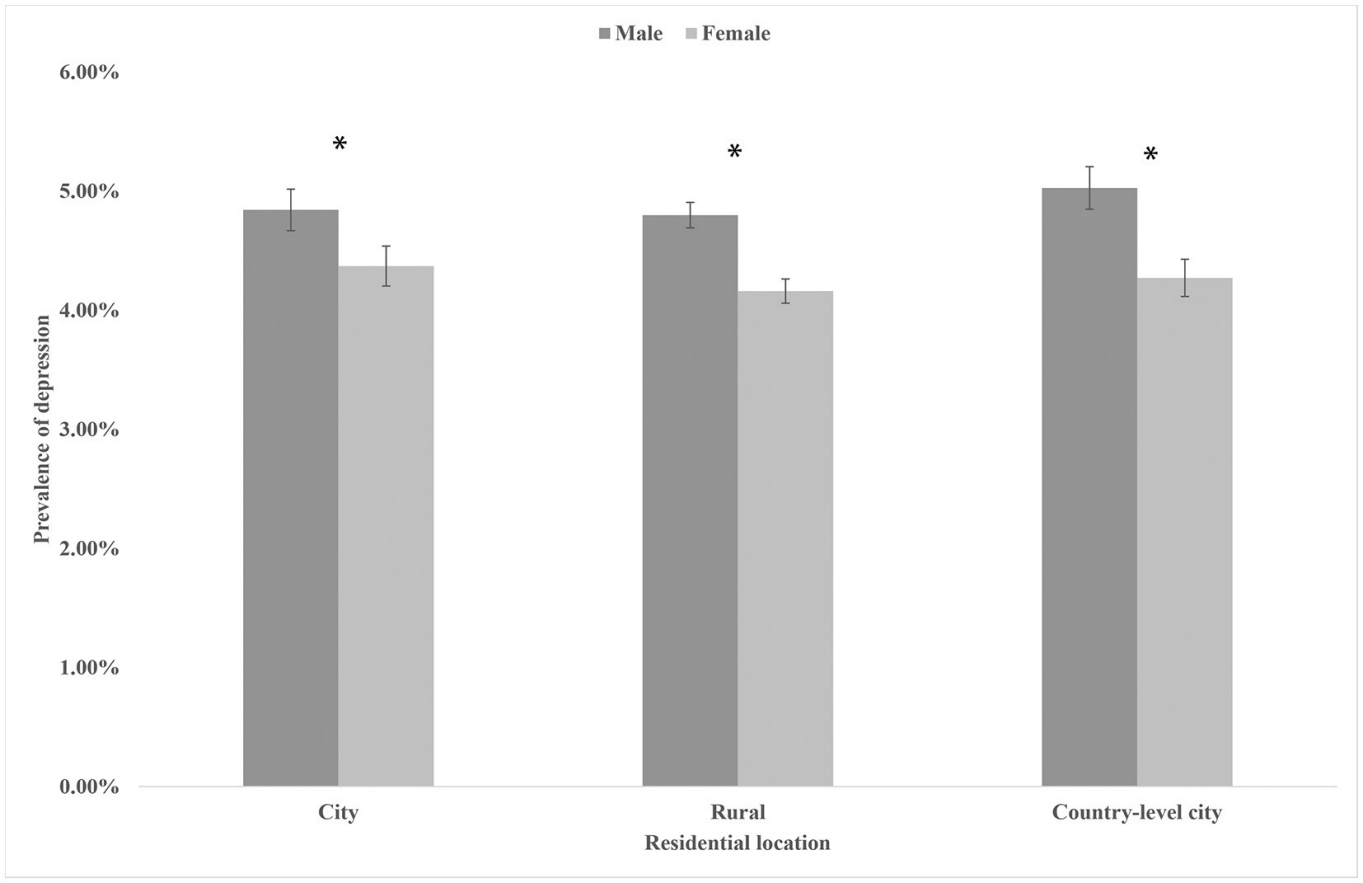
The prevalence of depression symptoms in participants by residential location and gender. * *P* < 0.05.

**Figure 2:**
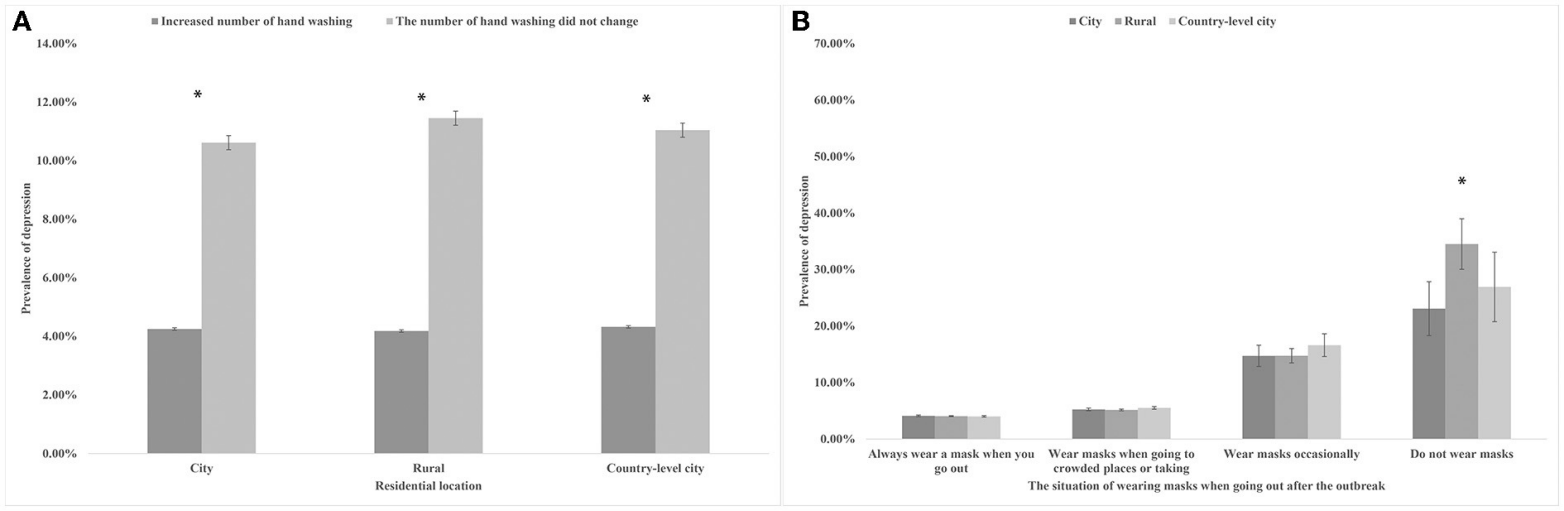
**(A)** The prevalence of depression symptoms in participants by degree of handwashing and residential location. * *P* < 0.05. **(B)** The prevalence of depression symptoms in participants by degree of wearing masks and residential location. * *P* < 0.05.

The overall depressive symptoms prevalence was 4.54% among college students during the COVID-19 pandemic in China. The prevalence of depressive symptoms in participants was shown in Figure 1 by gender and home region. Male participants living in country-level cities had the highest prevalence of depressive symptoms at 5.03%. Women who resided in rural areas had the lowest prevalence of depressed symptoms, at 4.16%. Overall, the prevalence of depressive symptoms was higher among men than among women, whether in cities, rural, or county-level cities. Figure 2A showed that whether in cities, rural, or country-level cities, the prevalence of depressive symptoms among students who did not change the number of handwashing was higher than those who increased the number of handwashing. Figure 2B showed that the prevalence of participants who lived in rural who do not wear masks was higher than participants who lived in the country-level city who do not wear masks (34.51 vs. 26.92%), but the opposite was true in participants who wear masks occasionally, where participants who lived in the country-level city was higher than the participants who lived in the rural areas (16.62 vs. 14.75%).

### Depressive symptoms

**Figure 3:**
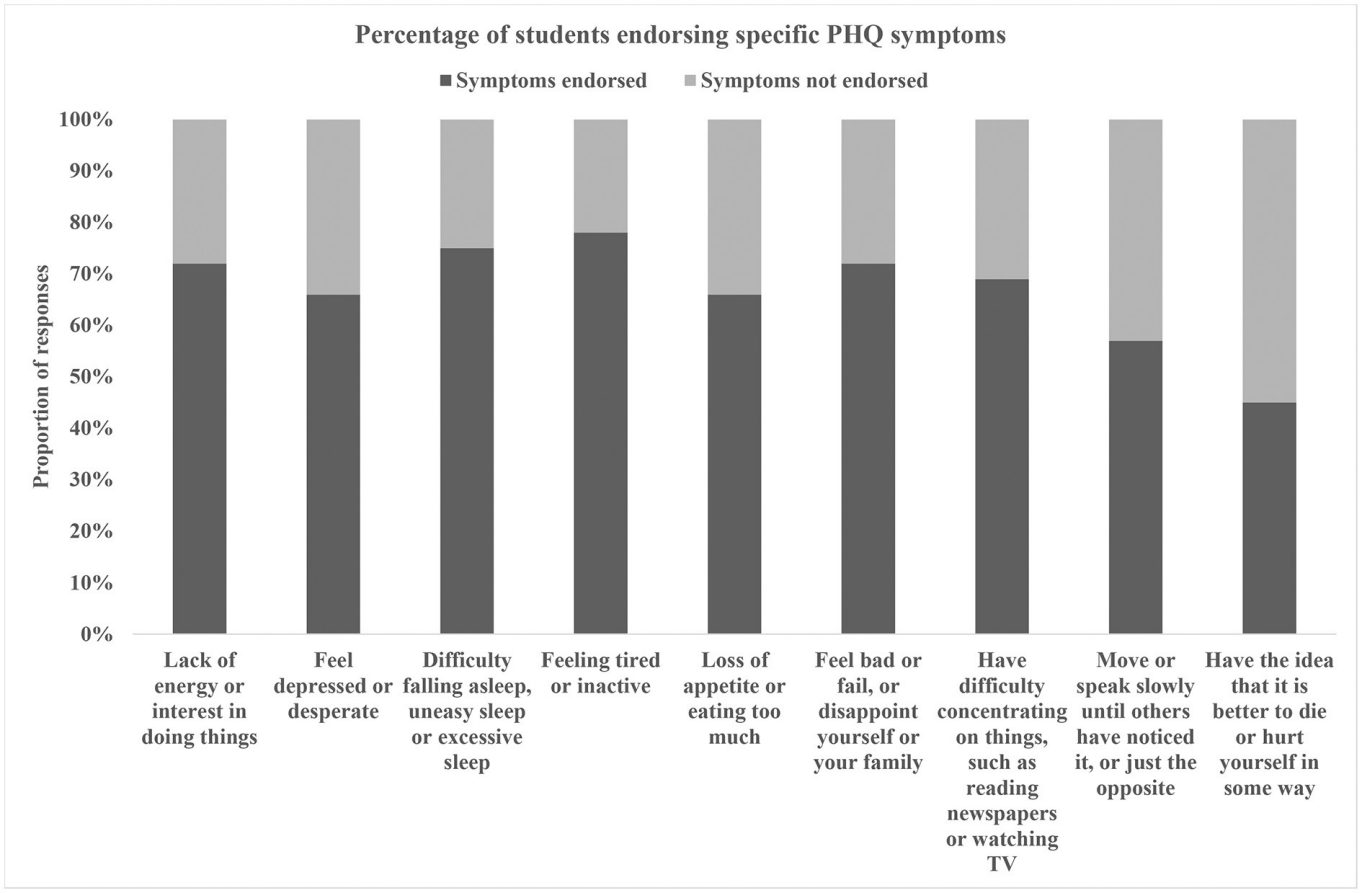
The proportion of nine PHQ symptoms in depression participants.

Table 2 shows the percentage of pupils with varying levels of depressive symptoms. The proportion of depressive symptoms from none to severe were 78.88, 15.78, 2.79, 1.67, and 0.88%, respectively. Mild depressive symptoms were most common. Compared to men, there were more women who had depressive symptoms (61.38%). But men were more likely to be moderately severe and severe. Obviously, the largest proportion of depressive symptoms occurred in rural (21.44%). The proportion of severe depressive symptoms was the highest (1.07%), although the city proportion of depressive symptoms was the lowest (20.29%). In participants with depressive symptoms, there was a variation in response rates across the nine PHQ symptoms, as shown in Figure 3. Obviously, feeling tired or inactive (78%) and difficulty falling asleep, uneasy sleep, or excessive sleep (75%) were the most common symptoms.

**Table 2:** The rate of different severities of depressive symptoms.

**Variables**	**None**		**Mild**		**Moderate**		**Moderately severe**		**Severe**	
	**n**	**%**	**n**	**%**	**n**	**%**	**n**	**%**	**n**	**%**
**Total**	110,630	78.88	22,129	15.78	3,923	2.80	2,341	1.67	1,236	0.88
**Gender**										
Male	56,456	40.25	9,752	6.95	1,807	1.29	1,322	0.94	786	0.56
Female	54,174	38.62	12,377	8.82	2,116	1.51	1,019	0.73	450	0.32
**Residential location**										
City	23,883	79.71	4,463	14.90	802	2.68	494	1.65	320	1.07
Rural	61,750	78.56	12,698	16.15	2,218	2.82	1,298	1.65	640	0.81
Country-level city	24,997	78.87	4,968	15.68	903	2.85	549	1.73	276	0.87

*Data were presented as mean (SD) normal distribution continuous variables and numbers (percentages) for categorical variables*.

### The positive or risk factors of depressive symptoms

Using multivariable logistic regression analysis, Table 3 showed the relationship between participant characteristics and depressive symptoms. Compared with men, women had 17% [OR 0.83 (95%CI: 0.79–0.88)] reduced odds of depressive symptoms. Meanwhile, after adjusted, students from rural had 8% reduced odds of anxiety [OR 0.92 (95%CI: 0.86–0.98)] compared to the cities. Compared with students with correct behavioral status about epidemic characteristics, students with incorrect behavioral status had higher odds of depressive symptoms. For example, students who make the number of hand-washing unchanged after the outbreak had 232% [OR 3.32 (95%CI: 3.05–3.61)] increased odds of depressive symptoms, compared with students who has the number of handwashing increased significantly after the outbreak. Similarly, compared with students who always wear a mask when they go out, students who wear masks when going to crowded places or taking public transport had 49% [OR 1.49 (95%CI: 1.41–1.57)] increased odds of depressive symptoms. Meanwhile, students who wear masks occasionally [OR 4.67 (95%CI: 4.02–5.42)] and students who do not wear masks [OR 11.26 (95%CI: 8.48–14.96)] had higher odds of depressive symptoms.

**Table 3:** Independent association of characteristics of study participants and depression during the COVID-19 epidemic in Henan province.

**Characteristics**	**Model 1**		**Model 2**	
	**OR (95%CI)**	***P* value**	**OR (95%CI)**	***P* value**
**Gender**				
Male	1.00 (ref)		1.00 (ref)	
Female	0.87 (0.82–0.91)	<0.05	0.83 (0.79–0.88)	<0.05
**Residential location**				
City	1.00 (ref)		1.00 (ref)	
Rural	0.97 (0.91–1.04)	0.38	0.92 (0.86–0.98)	<0.05
Country-level city	1.00 (0.93–1.08)	0.91	0.98 (0.91–1.06)	0.67
**Has the number of handwashing increased significantly after the outbreak**
Yes	1.00 (ref)		1.00 (ref)	
No	2.84 (2.61–3.08)	<0.05	3.32 (3.05–3.61)	<0.05
**The situation of wearing masks when going out after the outbreak**
Always wear a mask when you go out	1.00 (ref)		1.00 (ref)	
Wear masks when going to crowded places or taking public transport	1.31 (1.24–1.39)	<0.05	1.49 (1.41–1.57)	<0.05
Wear masks occasionally	4.14 (3.58–4.80)	<0.05	4.67 (4.02–5.42)	<0.05
Do not wear masks	9.76 (7.39–12.89)	<0.05	11.26 (8.48–14.96)	<0.05

*OR, odds ratio; CI, confidence interval.*

*Model 1: no adjustment.*

*Model 2: adjusted for gender, residential location, worried level, and fear level*.

## Discussion

This is a large-scale cross-sectional epidemiological investigation that took place in Henan Province. We investigated the prevalence of depressive symptoms among 140,259 college students during an outbreak of COVID-19. Our study showed that the prevalence of mild depressive symptoms and above among college students is 21.12%, which was lower than previous studies (37%) (36). Gender, residential location, and behavioral status were found to be associated with depressive symptoms among college students.

The overall prevalence of depressive symptoms in the present study was higher than in previous studies, which may be due to the introduction of epidemic prevention and control measures during the COVID-19 period (30). Some studies have found that female students have higher levels of depressive symptoms than male students (23, 37, 38). However, in contrast to previous findings (39), in our study, male students had more depressing symptoms than female students. Previous research has found similar differences in depressive symptoms between male and female students (40, 41). This could be due to the fact that in China, conventional gender roles and divisions still exist (42). During China's COVID-19, Chinese men as family pillars must bear more psychological pressure to ensure adequate supply and family safety, such as taking high-risk family matters. This differs from studies in other countries (43). At the same time, in the case of COVID-19, the lack of coping strategies will exacerbate male mental health problems (44).

Meanwhile, participants in our study who lived in rural areas had the highest proportion of depressed symptoms (Table 2). Differences in education, family income, medical insurance, and other social-culture factors could explain the disparity between city and rural residents (45). Students from rural areas are more likely to have come from poor families and are regarded as having a lower social status, resulting in disadvantages (46). Lower economic development levels in rural have reduced the ability to solve the problems brought by COVID-19.

Correct behavioral status was identified as a protective factor for our study. Even if only 0.17% of participants stated that they did not wear a mask during the outbreak, they were 1,026% more likely to exhibit depressive symptoms. Depressive symptoms were substantially more common among pupils who had an inappropriate behavioral status. This research emphasizes the need for educational intervention and the dissemination of accurate information. Our research found that knowing the correct type of mask was linked to a lower likelihood of depressive symptoms among college students. Wearing masks has been proven in patients with subclinical or mild COVID-19 to minimize the production of salivary and respiratory droplets (47). Wearing a mask, which is likely the most effective psychological sign for the general public, is necessary as a vital piece of personal protection equipment (48). Furthermore, our findings on COVID-19 health beliefs and face mask use point to some critical health literacy challenges. Because effective communicable disease prevention necessitates individuals to avoid activities that pose a high risk of infection and comprehending the rationale behind suggestions calling for societal responsibility to combat the pandemic, high levels of health literacy are critical (49). Europeans, on the other hand, have mixed feelings about face masks for cultural reasons. The need to utilize them is typically difficult for Europeans to accept (50). It also recommends that we should boost COVID-19 knowledge promotion, particularly in the behavioral status (51). Students can have a better understanding of COVID-19 through public awareness and education, allowing them to protect themselves from COVID-19-related depressed symptoms by practicing good hygiene, wearing a mask, exercising, and eating well (30).

Furthermore, we differentiated the severity of depression symptoms. The findings revealed that the majority of college students had mild depressive symptoms, with only a minority having moderate-to-severe depressive symptoms. It is worth noting that among the students who have depressive symptoms, feeling tired or inactive (78%) and difficulty falling asleep, uneasy sleep, or excessive sleep (75%) are the most common symptoms. As a result, we proposed that the health department establish an online psychological intervention platform where students can seek online psychological assistance if they are experiencing the two symptoms listed earlier (30).

To our knowledge, this is a large sample study of the prevalence of depressive symptoms among college students. Second, to diagnose depression symptoms, we employed the PHQ-9 standardized questionnaire. Finally, to make our results more realistic, we removed participants who did not match the study's conditions. However, some limitations should be recognized when discussing our findings. First, although we corrected several covariates, some potential confounding effects cannot be excluded. Second, because the study is cross-sectional, it is unable to draw inferences regarding the cause-and-effect linkages between the variables. Third, the behavioral status represents the participants' awareness of the COVID-19 pandemic characteristics, although its effectiveness cannot be guaranteed. Finally, because the participants in this study were college students, our findings may not apply to students in other grades.

## Conclusion

In conclusion, the prevalence of depressive symptoms among college students was not optimal during the COVID-19 pandemic normalization, particularly among students residing in the rural areas. Furthermore, in the follow-up work, factors such as gender, home location, and behavioral status should be evaluated as part of the overall management of depressive symptoms. These findings imply that in order to prevent COVID-19, governments should pay attention to college' student's mental health, and we should increase the COVID-19 knowledge promotion, particularly in behavioral status.

## Data availability statement

The raw data supporting the conclusions of this article will be made available by the authors, without undue reservation.

## Ethics statement

The studies involving human participants were reviewed and approved by Ethics Committee of the Zhengzhou University (ZZUIRB2021-118). The patients/participants provided their written informed consent to participate in this study. Written informed consent was obtained from the individual(s) for the publication of any potentially identifiable images or data included in this article.

## Author contributions

Conceptualization: XLu and QX. Data curation, visualization, and writing—original draft: XLu. Investigation: KF, JW, DW, XW, XLo, and HL. Writing—review and editing: QX, KF, CWa, CWu, and ZM. All authors contributed to the article and approved the submitted version.

## Funding

This work was supported by the National Natural Science Foundation of China (82041021).

## Conflict of interest

The authors declare that the research was conducted in the absence of any commercial or financial relationships that could be construed as a potential conflict of interest.

## Publisher's note

All claims expressed in this article are solely those of the authors and do not necessarily represent those of their affiliated organizations, or those of the publisher, the editors and the reviewers. Any product that may be evaluated in this article, or claim that may be made by its manufacturer, is not guaranteed or endorsed by the publisher.
